# Implementation of Objective PASC-Derived Taxon Demarcation Criteria for Official Classification of Filoviruses

**DOI:** 10.3390/v9050106

**Published:** 2017-05-11

**Authors:** Yīmíng Bào, Gaya K. Amarasinghe, Christopher F. Basler, Sina Bavari, Alexander Bukreyev, Kartik Chandran, Olga Dolnik, John M. Dye, Hideki Ebihara, Pierre Formenty, Roger Hewson, Gary P. Kobinger, Eric M. Leroy, Elke Mühlberger, Sergey V. Netesov, Jean L. Patterson, Janusz T. Paweska, Sophie J. Smither, Ayato Takada, Jonathan S. Towner, Viktor E. Volchkov, Victoria Wahl-Jensen, Jens H. Kuhn

**Affiliations:** 1 BIG Data Center, Beijing Institute of Genomics, Chinese Academy of Sciences, Beijing 100101, China; baoym@big.ac.cn; 2 Department of Pathology and Immunology, Washington University School of Medicine, St. Louis, MO 63110, USA; GAmarasinghe@path.wustl.edu; 3 Center for Microbial Pathogenesis, Institute for Biomedical Sciences, Georgia State University, Atlanta, GA 30302-3965, USA; cbasler@gsu.edu; 4 United States Army Medical Research Institute of Infectious Diseases, Fort Detrick, Frederick, MD 21702-5011, USA; sina.bavari.civ@mail.mil (S.B.); john.m.dye1.civ@mail.mil (J.M.D.); 5 Department of Pathology, The University of Texas Medical Branch, Galveston, TX 77555-0144, USA; alexander.bukreyev@utmb.edu; 6 Department of Microbiology and Immunology, Albert Einstein College of Medicine, Bronx, New York, NY 10461, USA; kartik.chandran@einstein.yu.edu; 7 Institute of Virology, Philipps University Marburg, 35032 Marburg, Germany; Dolnik@staff.uni-marburg.de; 8 Department of Molecular Medicine, Mayo Clinic, Rochester, MN 55905, USA; Ebihara.Hideki@mayo.edu; 9 World Health Organization, 1211 Geneva, Switzerland; formentyp@who.int; 10 Public Health England, Porton Down, Wiltshire, Salisbury SP4 0JG, UK; Roger.Hewson@phe.gov.uk; 11 Department of Microbiology, Immunology & Infectious Diseases, Université Laval, Quebec City, QC G1V 0A6, Canada; gary.kobinger@crchudequebec.ulaval.ca; 12 Centre International de Recherches Médicales de Franceville, Institut de Recherche pour le Développement, BP 769 Franceville, Gabon; eric.leroy@ird.fr; 13 Department of Microbiology and National Emerging Infectious Diseases Laboratories, Boston University School of Medicine, Boston, MA 02118, USA; muehlber@bu.edu; 14 Novosibirsk State University, Novosibirsk, Novosibirsk Oblast, Russia 630090; netesov.s@nsu.ru; 15 Department of Virology and Immunology, Texas Biomedical Research Institute, San Antonio, TX 78245-0549, USA; jpatters@TxBiomed.org; 16 Center for Emerging and Zoonotic Diseases, National Institute for Communicable Diseases of the National Health Laboratory Service, Sandringham-Johannesburg 2131, Gauteng, South Africa; januszp@nicd.ac.za; 17 Chemical, Biological and Radiological Division, Defence Science and Technology Laboratory, Porton Down, Salisbury, Wiltshire SP4 0JQ, UK; SJSMITHER@mail.dstl.gov.uk; 18 Division of Global Epidemiology, Hokkaido University Research Center for Zoonosis Control, Sapporo 001-0020, Japan; atakada@czc.hokudai.ac.jp; 19 Viral Special Pathogens Branch, Division of High-Consequence Pathogens Pathology, National Center for Emerging and Zoonotic Infectious Diseases, Centers for Disease Control and Prevention, Atlanta, GA 30329-4027, USA; jit8@cdc.gov; 20 Molecular Basis of Viral Pathogenicity, CIRI, INSERM U1111-CNRS UMR5308, Université de Lyon, Université Claude Bernard Lyon 1, Ecole Normale Supérieure de Lyon, Lyon 69007, France; viktor.volchkov@inserm.fr; 21 National Biodefense Analysis and Countermeasures Center, Fort Detrick, Frederick, MD 21702, USA; victoria.jensen@nbacc.dhs.gov; 22 Integrated Research Facility at Fort Detrick, National Institute of Allergy and Infectious Diseases, National Institutes of Health, Frederick, MD 21702, USA

**Keywords:** cuevavirus, Ebola, ebolavirus, *Filoviridae*, filovirus, marburgvirus, *Mononegavirales*, virus taxonomy, virus classification, ICTV

## Abstract

The mononegaviral family *Filoviridae* has eight members assigned to three genera and seven species. Until now, genus and species demarcation were based on arbitrarily chosen filovirus genome sequence divergence values (≈50% for genera, ≈30% for species) and arbitrarily chosen phenotypic virus or virion characteristics. Here we report filovirus genome sequence-based taxon demarcation criteria using the publicly accessible PAirwise Sequencing Comparison (PASC) tool of the US National Center for Biotechnology Information (Bethesda, MD, USA). Comparison of all available filovirus genomes in GenBank using PASC revealed optimal genus demarcation at the 55–58% sequence diversity threshold range for genera and at the 23–36% sequence diversity threshold range for species. Because these thresholds do not change the current official filovirus classification, these values are now implemented as filovirus taxon demarcation criteria that may solely be used for filovirus classification in case additional data are absent. A near-complete, coding-complete, or complete filovirus genome sequence will now be required to allow official classification of any novel “filovirus.” Classification of filoviruses into existing taxa or determining the need for novel taxa is now straightforward and could even become automated using a presented algorithm/flowchart rooted in RefSeq (type) sequences.

## 1. Introduction

The family *Filoviridae*, one of eight families in the order *Mononegavirales* [1], has eight members assigned to seven species included in three genera (Table 1) [2,3,4].

Traditionally, the eight currently recognized filoviruses have been classified using phenotypic characteristics of virions and/or partial filovirus genome sequences [5,6,7]. Sequence-based filovirus taxon demarcation criteria (nucleotide and amino acid sequence identity values and/or phylogenies) were officially introduced as additional demarcation criteria in 2000 [8] and further refined thereafter [9]. Yet, true filovirus genome sequence-based taxon demarcation was only introduced in 2011. At that time, the International Committee on Taxonomy of Viruses (ICTV) *Filoviridae* Study Group decided arbitrarily that marburgvirus genomes differ from ebolavirus genomes by ≥50% and that ebolavirus species are differentiated on the basis of glycoprotein (*GP*) gene sequence differences (≥30%) or genome sequence differences (≥30%) [3]. These values were used to develop a decision algorithm/flowchart for filovirus taxon assignment that could guide filovirus classification [10]. In 2012, two pairwise sequence comparison methods, PAirwise Sequence Comparison (PASC) and DivErsity pArtitioning by hieRarchical Clustering (DEmARC), confirmed that the then official filovirus taxonomy (identical to the current one shown in Table 1) is justified, but that the 50% and 30% values ought to be adjusted objectively based on the PASC and/or DEmARC results [11,12]. Both analyses were based on the available ≈50 near-complete, coding-complete or complete filovirus genomes (see [13,14] for nomenclature) in the US National Center for Biotechnology Information (NCBI, Bethesda, MD, USA) GenBank database. Yet, at the time it was unclear whether the ICTV would accept classification of viruses based on sequence analysis alone.

In 2017, the ICTV members reached a consensus together with other experts that “the development of a robust framework for sequence-based virus taxonomy is indispensable for the comprehensive characterization of the global virome” [15]. Under proper oversight by, for instance, ICTV Study Groups, virus classification criteria can now be based on measurable objective criteria inferable only from viral genome sequence data. Thus, using automatic classification algorithms is possible.

The number of GenBank-deposited near-complete, coding-complete, and complete filovirus genome sequences has increased substantially in recent years (from the ≈50 in 2012 to ≈1400 at the time of writing in 2017). We analyzed these sequences using PASC, a method that can be easily used by any scientist using an open-access software platform [16,17,18]. We created inferred objective filovirus taxon demarcation criteria and updated the algorithm/flowchart for filovirus taxon assignment using the recently decided type filovirus sequences (NCBI RefSeq database sequences) [10] as starting points.

## 2. Materials and Methods

All 1404 near-complete, coding-complete, or complete filovirus genomes available from GenBank (NCBI, Bethesda, MD, USA) on 04/16/2017 were downloaded from the NCBI viral genomes resource [19]. Redundant filovirus genome sequences (here defined as sequences with PASC identities >99.5%) were removed, leaving 112 filovirus genome sequences for further analysis [20]. PASC analysis was performed with those 112 genome sequences as previously described [18] using the open-access PASC tool (NCBI). The new taxon demarcation algorithm/flowchart was developed based on the previously developed chart presented in [10] using type filoviruses [4] and type filovirus genome sequences (RefSeq, NCBI) [10].

## 3. Results

**Figure 1:**
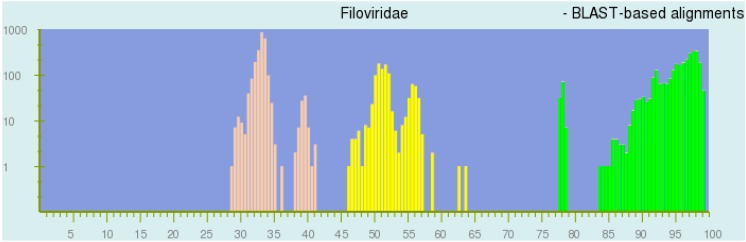
Screenshot of the US National Center for Biotechnology Information (NCBI) PAirwise Sequence Comparison (PASC) tool result after comparing 112 distinct near-complete, coding-complete or complete filovirus genome sequences. Brown bars represent genome pairs assigned to (three) different genera; yellow bars represent genome pairs assigned to (seven) separate species; and green bars represent genome pairs assigned to the same species. BLAST: Basic Local Alignment Search Tool.

PASC analysis of 112 filovirus near-complete, coding-complete, or complete genome sequences revealed clear clustering into three higher ranks (genera), with two of those genera including single species and one genus including five species (visualized in Figure 1).

Unblinding of input sequences revealed the three genera and seven species to correspond to those already established and depicted in Table 1, raising confidence in PASC as a method to adequately recreate current knowledge on filovirus diversity. However, the analysis indicated an ideal genus demarcation threshold range of 55–58% sequence divergence rather than the currently used 50% threshold and an ideal species demarcation threshold range of 23–36% rather than the currently used 30% threshold.

## 4. Discussion

**Figure 2:**
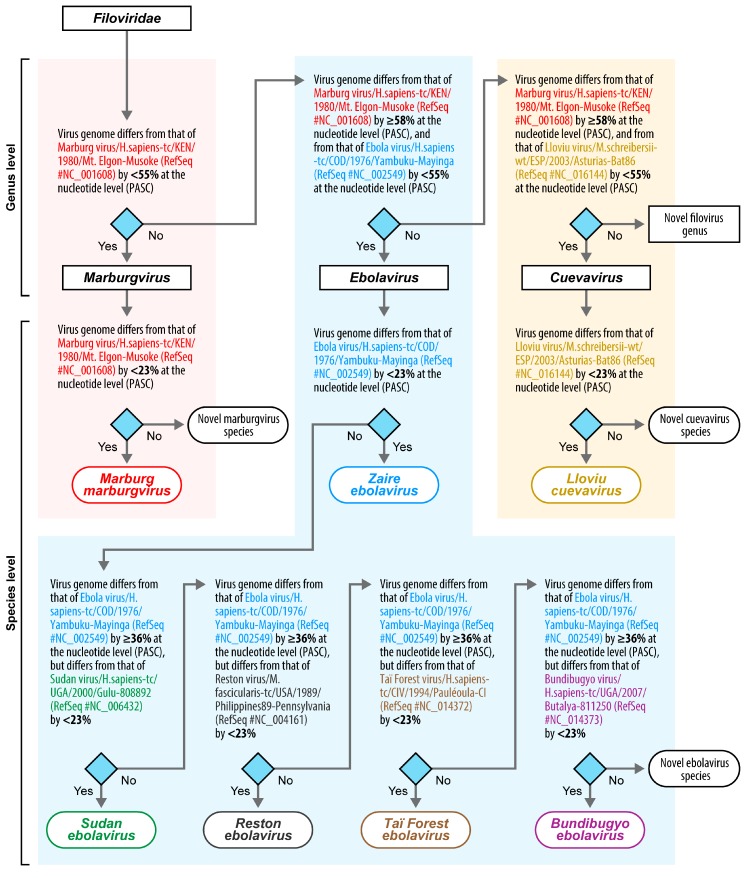
Algorithm/flow chart for filovirus classification based on genomics sequence information (modified from [10]) and PASC-derived sequence demarcation criteria. A putative filovirus genome of interest is compared to the type filovirus RefSeq genome sequence (i.e., that of Marburg virus/H.sapiens-tc/KEN/1980/Mt. Elgon-Musoke [10]) and then sequentially moved through the process until its proper placement in a species is revealed. If the sequence comparison reveals the need for the creation of a novel genus and/or species, official taxonomic proposals ought to be submitted to the ICTV.

Using the new filovirus taxon demarcation criteria established here using PASC, the earliest discovered filovirus (Marburg virus; MARV) as the type virus for the family *Filoviridae* [4], the RefSeq MARV genome sequence as the MARV type sequence, and the remaining filovirus RefSeq genome sequences as additional anchor points, we created a filovirus classification decision matrix in form of an algorithm/flowchart (Figure 2). Using the NCBI PASC tool and Figure 2, any user can now quickly assess whether a novel filovirus sequence of interest represents a filovirus already classified in one of the established filovirus taxa or whether establishment of a new taxon/new taxa may be necessary. PASC requires at least near-complete or coding-complete genome input sequences. Therefore, the ICTV *Filoviridae* Study Group decided that moving forward, at least a coding-complete filovirus genome sequence will be minimally required for filovirus classification into novel filovirus taxa. Partial filovirus-like nucleic acids, for instance, those recently discovered in Chinese bats [21,22], may point towards the existence of novel filoviruses but will not suffice for official recognition of novel filoviruses or establishment of novel filovirus taxa. The Study Group recommends that such sequences be referred to as “filovirus-like sequences” and not as “filoviruses.” Likewise, a virus for which a partial filovirus-like sequence information exists ought to be referred to as a “putative filovirus” until at least coding-complete genome sequence information is available. 

Importantly, PASC analysis followed by use of the algorithm/flowchart (Figure 2) alone does not constitute official classification, and the Study Group sees PASC results as highly informative, but not binding. Thus, if the PASC algorithm/flowchart indicates the need for a novel filovirus genus and/or species to a user analyzing a particular sequence, the user should follow the official pathway for ICTV classification starting with submission of an official taxonomic proposal (TaxoProp [23]). The user is recommended to engage with the ICTV *Filoviridae* Study Group as early as possible during that process. The Study Group and ICTV will evaluate all available data on a particular putative filovirus (e.g., host information, disease phenotype, biophysical properties of virions) and make their decisions accordingly. Phylogenetic results obtained with methods more sophisticated than PASC are always desired and may ultimately overrule PASC results.

## Figures and Tables

**Table 1:** Official filovirus taxonomy endorsed by the 2015–2017 International Committee on Taxonomy of Viruses (ICTV) *Filoviridae* Study Group and accepted by the ICTV.

Current Taxonomy and Nomenclature
Order *Mononegavirales* Family *Filoviridae* Genus *Marburgvirus* Species *Marburg Marburgvirus* Virus 1: Marburg virus (MARV) Virus 2: Ravn virus (RAVV) Genus *Ebolavirus* Species *Bundibugyo ebolavirus* Virus: Bundibugyo virus (BDBV) Species *Reston ebolavirus* Virus: Reston virus (RESTV) Species *Sudan ebolavirus* Virus: Sudan virus (SUDV) Species *Taï Forest ebolavirus* Virus: Taï Forest virus (TAFV) Species *Zaire ebolavirus* Virus: Ebola virus (EBOV) Genus *Cuevavirus* Species *Lloviu cuevavirus* Virus: Lloviu virus (LLOV)
